# Incorporating a molecular antenna in diatom microalgae cells enhances photosynthesis

**DOI:** 10.1038/s41598-021-84690-z

**Published:** 2021-03-04

**Authors:** Gabriella Leone, Gabriel De la Cruz Valbuena, Stefania Roberta Cicco, Danilo Vona, Emiliano Altamura, Roberta Ragni, Egle Molotokaite, Michela Cecchin, Stefano Cazzaniga, Matteo Ballottari, Cosimo D’Andrea, Guglielmo Lanzani, Gianluca Maria Farinola

**Affiliations:** 1 Dipartimento Di Chimica, Università Degli Studi Di Bari “Aldo Moro”, via Orabona 4, 70126 Bari, Italy; 2 Center for Nano Science and Technology, Istituto Italiano Di Tecnologia, via Pascoli 70/3, 20133 Milano, Italy; 3 Dipartimento Di Fisica, Politecnico Di Milano, Piazza Leonardo da Vinci 32, 20133 Milano, Italy; 4 Dipartimento Di Chimica, CNR-ICCOM, Università Degli Studi Di Bari “Aldo Moro”, via Orabona 4, 70126 Bari, Italy; 5 Dipartimento Di Biotecnologie, Università Degli Studi Di Verona, Ca’ Vignal 1, 37134 Verona, Italy

**Keywords:** Environmental biotechnology, Chemical ecology, Environmental chemistry, Light responses, Photosynthesis

## Abstract

Diatom microalgae have great industrial potential as next-generation sources of biomaterials and biofuels. Effective scale-up of their production can be pursued by enhancing the efficiency of their photosynthetic process in a way that increases the solar-to-biomass conversion yield. A proof-of-concept demonstration is given of the possibility of enhancing the light absorption of algae and of increasing their efficiency in photosynthesis by in vivo incorporation of an organic dye which acts as an antenna and enhances cells’ growth and biomass production without resorting to genetic modification. A molecular dye (Cy5) is incorporated in *Thalassiosira weissflogii* diatom cells by simply adding it to the culture medium and thus filling the orange gap that limits their absorption of sunlight. Cy5 enhances diatoms’ photosynthetic oxygen production and cell density by 49% and 40%, respectively. Cy5 incorporation also increases by 12% the algal lipid free fatty acid (FFA) production versus the pristine cell culture, thus representing a suitable way to enhance biofuel generation from algal species. Time-resolved spectroscopy reveals Förster Resonance Energy Transfer (FRET) from Cy5 to algal chlorophyll. The present approach lays the basis for non-genetic tailoring of diatoms’ spectral response to light harvesting, opening up new ways for their industrial valorization.

## Introduction

Drastic changes in climate and reduction in the availability of raw chemical materials are drawing academic and industrial research, as a matter of considerable urgency, towards photosynthetic organisms as living factories for the large-scale production of fuels and active chemical products. Phytoplankton have great industrial potential in this context, which includes diatoms, the major group of microalgae responsible for ocean pH, worldwide carbon recycling and atmospheric CO_2_ regulation^1^. Diatoms are envisioned as a valuable energy and food source for the near future, potentially producing more biomass per unit of land area than terrestrial organisms^2^. These unicellular eukaryotic microalgae^3^ are the main oxygen producers in marine ecosystems^4,5^ and control the Earth’s carbon cycle, as they are responsible for ~ 40% of total organic carbon produced yearly in seawater^6,7^. Their excellent lipid-accumulation properties make diatoms promising candidates for large-scale production of biofuels^8^. Further scientific interest in diatoms centers on their suitability for application in biomedicine^9–12^ and photonics^13–15^ given their mesoporous silica cell walls (frustules), whose hierarchically organized nanostructure has functions linked to cell protection from predators and harmful solar wavelengths.

Despite the variety of applications, the high costs of large-scale cultivation have so far restricted diatoms’ suitability for industrial production^16^. A possible way of circumventing this issue is to enhance their growth-improving photosynthetic efficiency that increases biomass and CO_2_ fixation^17^. In principle, photosynthesis can be enhanced by modifying external parameters such as CO_2_ concentration, light intensity^18^ or algal excitation wavelengths^19,20^ but these procedures face several limitations including cost of artificial illumination, change of the natural light availability, possible photoinhibition phenomena occurring at high irradiances and CO_2_ trapping in the liquid phase^21^. Photosynthetic efficiency can also be enhanced by genetic modification of microalgae, for example by inhibiting their photoprotective mechanisms that limit light absorption^22–24^. Genetic modification can overcome microalgal non-photochemical quenching (NPQ) by inhibition of genes that normally reduce photosynthetic efficiency under high light intensities. Genetic modification has been recently used to enhance photosynthesis of microalgae cells also by enlarging their sunlight absorption capability^25^. Fu et al*.* genetically expressed the enhanced green fluorescent protein (eGFP) in *Phaeodactylum Tricornutum* diatoms, with eGFP acting as a chromophore capable of absorbing light in solar spectral regions where algal absorption is otherwise limited and transferring the collected light energy to the pigment-protein complexes of the photosystem units. This approach favored photochemical reactions, allowing to outperform the wild-type parental microalgal strain in biomass production rate under outdoor simulated sunlight conditions^25^.

Nevertheless, genetic strategies require expensive tools, not straightforward experimental procedures and are still limited to a few genetically fully-sequenced species of diatoms^26–28^, which inevitably prevents their current general industrial application.

In vivo incorporation of tailored organic dyes into microalgae by simply adding them as photoactive nutrients into cells culture can, in principle, constitute an alternative photosynthesis-enhancing approach in living algal cells, promoting light harvesting without the need for genetic modification^29–32^. In last years, organic dyes were used to demonstrate the possibility to enhance photosynthesis in green microalgae such as *Chlorella sorokiniuna*^33^, *Dunaliella salina*^34^ and *Nannochloropsis gaditana*^35^: in particular, solutions of spectral shifting dyes were located in external cavities surrounding the microalgal photo-bioreactors, thus demonstrating that proximity of dyes solution kept separated from the algal culture can ensure transfer of the energy harvested by the dyes to algal cells without altering cells viability. However, this approach requires a complex and expensive design of photo-bioreactors since direct contact of dyes with cell cultures is avoided to preserve cells, this being incompatible with large scale applications.

Alternative photosynthesis-enhancing strategies, which in principle are more profitable in terms of scalability, resort to the incorporation of spectral shifting dyes into living algal cells. The dyes used must fulfill certain requirements: (*i*) irrelevant toxicity for the target living organisms, (*ii*) light absorption and emission properties suitable for energy transfer to algal photosystems, and (*iii*) amphiphilic chemical structures that are both dispersible in water and easily incorporated into cells. Current literature gives a few examples of studies relating to the effects on photosynthesis of the in vivo incorporation of organic dyes in diatoms cells. In particular, rhodamines have shown efficiency in penetrating diatom cells and staining both cell wall and cytoplasm^36^, but they were found to be toxic for various species such as *Coscinodiscus granii* and *wailesii*^37^. Their toxicity was also demonstrated for plants and fungal cells^38,39^, and the suitability of rhodamines was thus restricted to enhancing the efficiency of photosynthetic complexes extracted from photosynthetic organisms^40^.Recently, in vivo incorporation of a BODIPY (dipyrrometheneborondifluoride) dye into diatom microalgae has been shown to increase diatoms’ biomass rapidly in short-term cultivation but a decrease in cell population was observed 24 h after adding the dye, revealing overall harmful effects on diatom cultures rather than the expected beneficial cell proliferation^25^.

These studies thus confirm that, in principle, enhancement of algal photosynthesis can be pursued by the in vivo incorporation of dyes selected to extend algal absorption cross-sections, but the biocompatibility of incorporated dyes represents a crucial issue that must be overcome.

**Figure 1:**
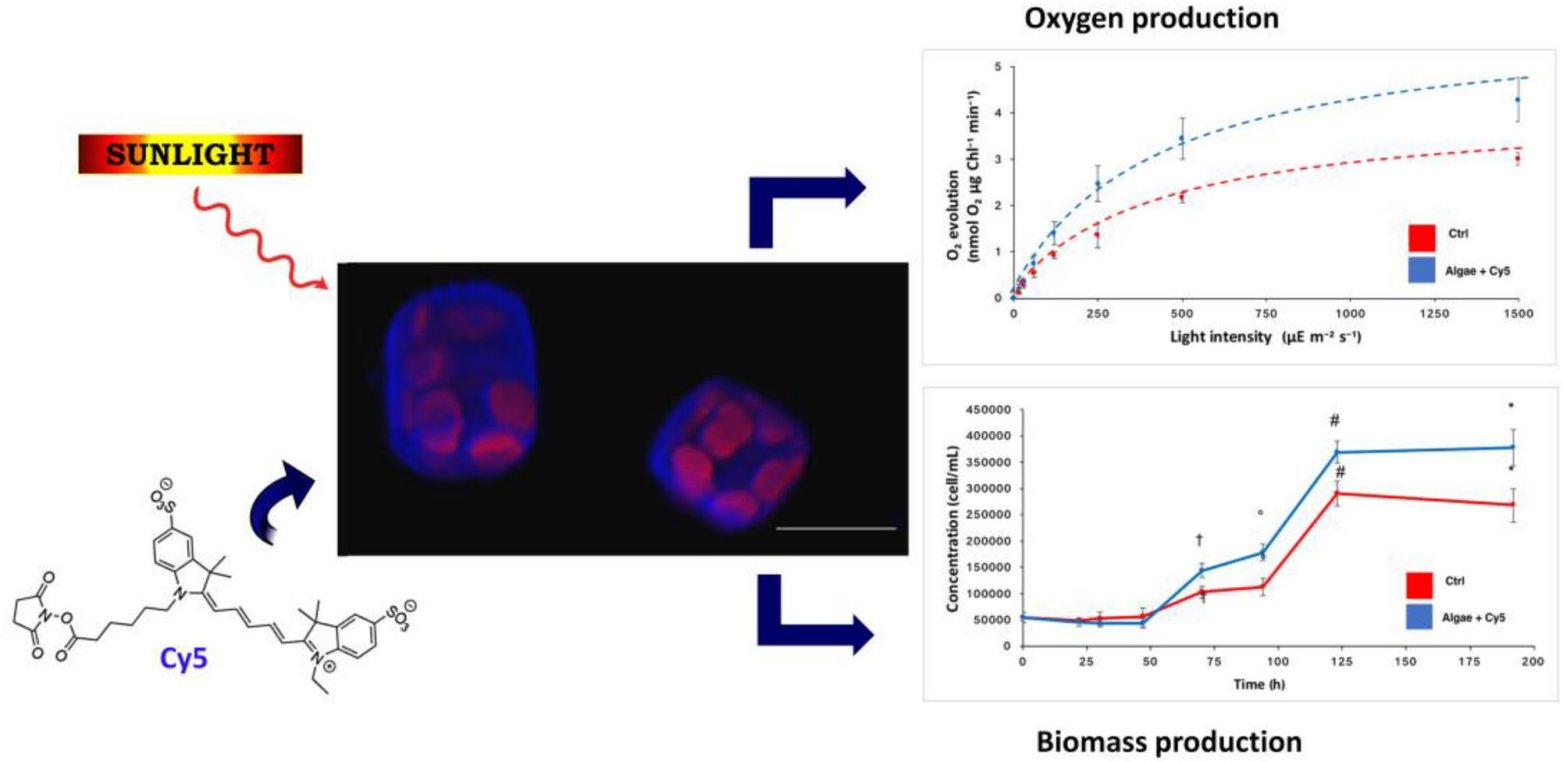
Effects of in vivo incorporation of the Cy5 antenna dye in *Thalassiosira weisflogii* diatoms. Scale bar of the confocal diatoms micrograph: 10 µm.

Here we demonstrate that, upon simple addition of a cyanine dye (Cy5, Fig. 1) to the culture medium of *Thalassiosira weissflogii* diatom cells, the dye incorporation into cells occurs without altering cells viability and induces an increase of light-dependent cell density (40%), biomass (23%) and lipid (12%) production, by improving diatoms’ spectral response to light harvesting with no need for genetic engineering. The enhancement of photosynthetic activity of Cy5 treated versus pristine diatoms was also confirmed by 49% increase of light-dependent oxygen production.

Confocal microscopy reveals that Cy5 rapidly penetrates into cells, being located both into frustules and organic protoplasm. Time-resolved spectroscopy indicates that energy transfer compatible with a FRET mechanism occurs between Cy5 and the chlorophyll a of living diatoms.

This proof-of-concept paves the way for the development of light harvesting dyes that, upon incorporation into living algae cells, can enhance photosynthesis and potentially favor large scale production of biomass to be used in different industrial sectors.

## Results

### The antenna dye

**Figure 2:**
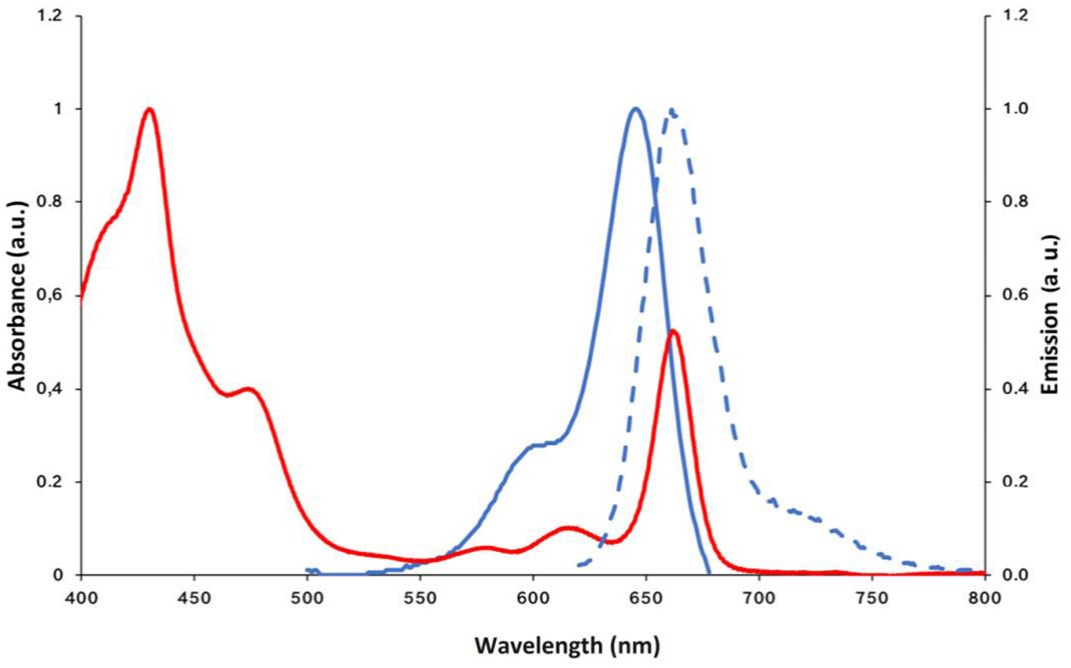
Absorption spectra of Cy5 (blue continuous line) and pigments (red line) extracted from *Thalassiosira weissflogii* diatoms and emission spectrum of Cy5 (dashed blue line) in seawater.

Cy5 is a cyanine dye selected as a model molecular antenna due to its (*i*) commercial availability, (*ii*) recognized suitability as an imaging tool for biomolecular systems, (*iii*) amphiphilic chemical structure (Fig. 1) favoring both molecular dispersion in aqueous media in the presence of biocompatible content of dimethylsulphoxide and molecular permeation through cell membranes. Moreover, (*iv*) light absorption and emission properties of Cy5 are suitable for light harvesting and energy transfer to chlorophylls of *Thalassiosira weissflogii* diatoms since Cy5 absorbs light in the range 570–650 nm and emits light at wavelengths (λ_max_: 660 nm) where the chlorophyll *a* absorption in diatoms cells is maximum (Fig. 2). In fact, the UV–vis absorption spectrum (red line in Fig. 2) of photosynthetic pigments extracted from *Thalassiosira weissflogii*, shows the major Chl *a* absorption peaks at 430 and 662 nm and less intense peaks due to xanthophylls (480 nm) and Chl *c* (580, 620 nm)^41,42^. The major Chl *c* absorption peak at 450 nm is weakly visible, being hidden by xanthophylls and Chl *a* absorption.

### Diatoms’ growth kinetics

**Figure 3:**
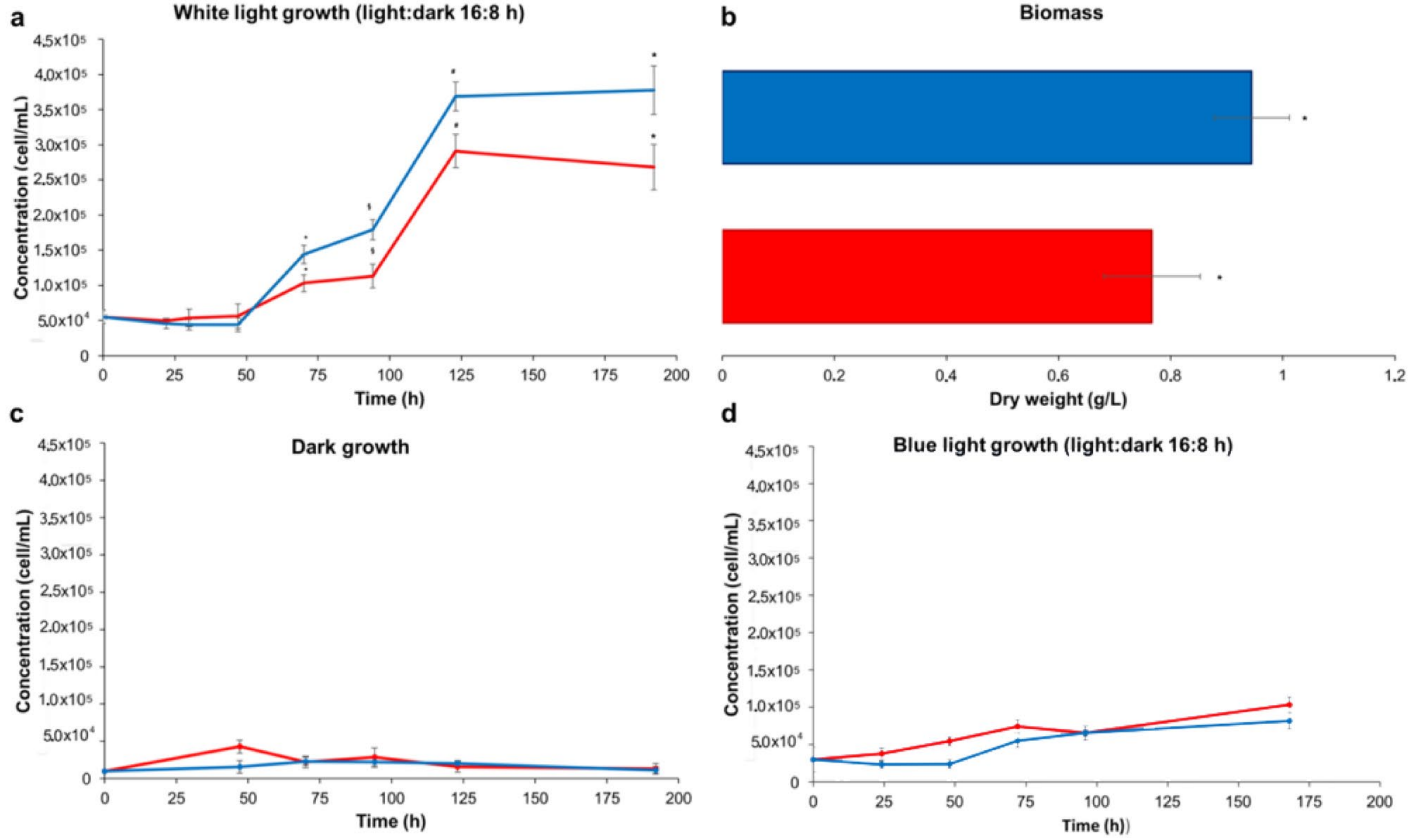
(**a**) Curves for diatom growth and (**b**) biomass recorded, under normal lighting conditions, in the presence (blue line) and absence (red line) of Cy5. Curves for diatom growth recorded under (**c**) darkness and (**d**) blue light excitation.

Diatoms’ growth in culture medium enriched with Cy5 (1 μM final concentration) was monitored under normal lighting conditions (light: dark 16 h:8 h, light intensity 70 µmol/m^2^ s) and compared to a reference sample of diatoms grown in the absence of the dye (Fig. 3a): in both cases, exponential growth started after 48 h, reaching a plateau at the 5th day. In particular, the concentration curve of Cy5 treated diatoms was steeper than in the control in the 48–70 h exponential growth period. After 8 days, the cell concentration of the sample treated with Cy5 exceeded the value observed for the control sample by 40%. The growth rate, calculated in the exponential phase between 48 and 70 h, was 0.9 ± 0.2 for algae grown with Cy5 and 0.4 ± 0.3 for the control.

We also observed a 23% increase in biomass dry weight (Fig. 3b) for algae grown for 8 days, under normal lighting conditions, in the presence of Cy5 versus the control.

Furthermore, the effect of Cy5 on diatoms’ lipid biosynthesis was explored. The lipid fraction of free fatty acids (FFA) was extracted from biomass of both pristine and Cy5 treated diatoms and FFA were subjected to esterification reaction to fatty acids methyl esters (FAME). This chemical conversion is commonly performed to increase sample volatility and improve resolution of GC–MS analysis. According to the literature, saturated and unsaturated C16 FFAs represent the most abundant portion of lipids extracted from *Thalassiosira weissflogii* diatoms^43^. As an effect of Cy5 algal incorporation, a 12% increase of C16 FAME lipidome mass was recorded by GC–MS (Supplementary Fig. S1a,b).

To check that the increase of algal growth and biomass was effectively related to the photosynthetic enhancement^17^, we performed the same experiments in absence of light, inhibiting photosynthesis (Fig. 3c). In this case, the growth curve of algae incubated with Cy5 did not differ from the control growth curve, thus confirming that cell growth and biomass production are dependent on dye photoexcitation and ruling out any possible effects of Cy5 in non-photosynthetic related metabolic pathways. As an additional control experiment, diatoms were grown in the presence of Cy5 and the culture excited with blue light (410–450 nm), which allowed algal photosynthesis to occur but excluded the contribution of Cy5 which is not excited at that wavelength (Fig. 3d). In this case, too, Cy5 did not affect algal growth with respect to the control.

### Photosynthetic activity

**Figure 4:**
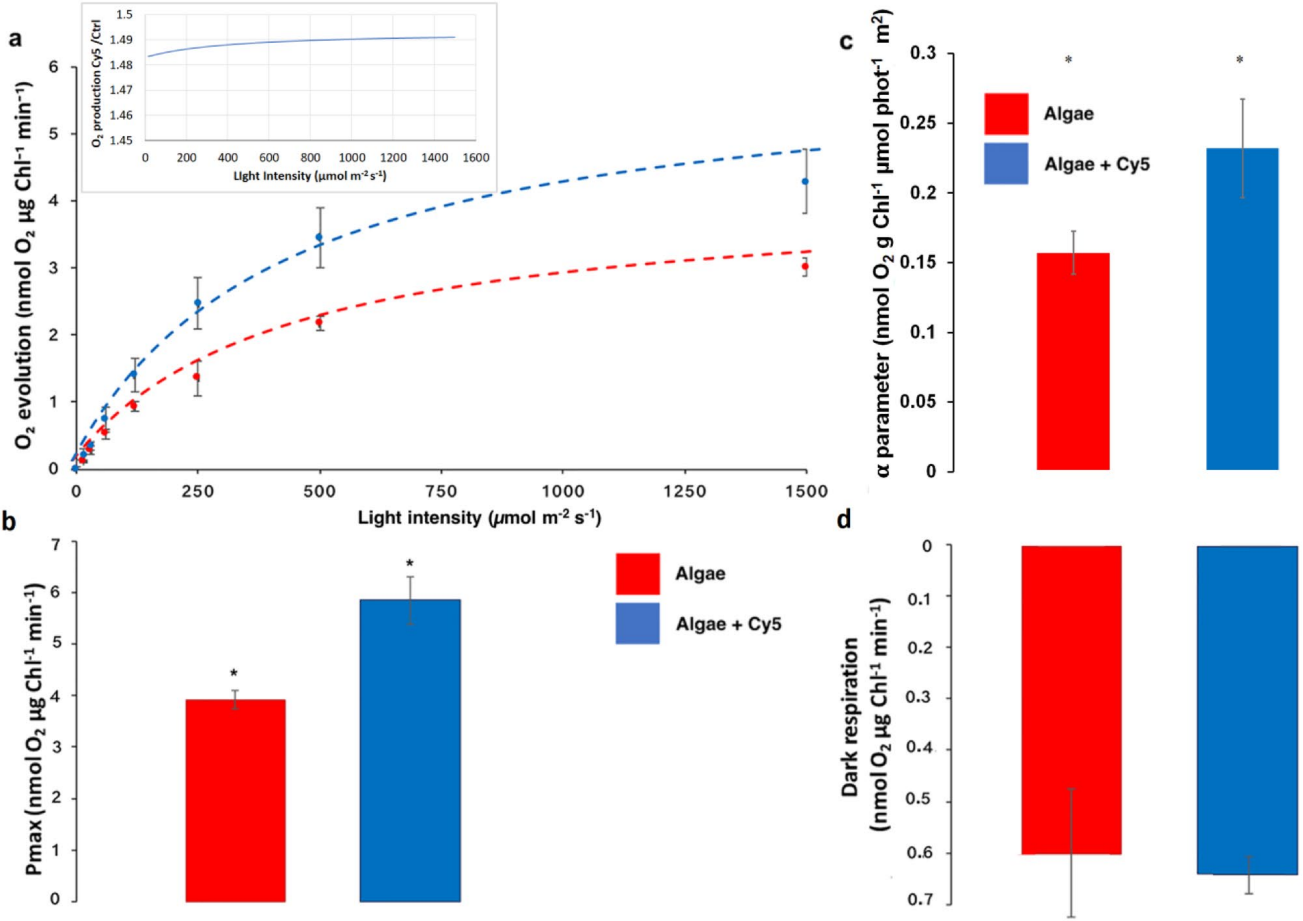
Light response curves. (**a**) Algae oxygen evolution rates at different actinic lights in the presence (blue line) and absence (red line) of Cy5. (**b**) Pmax: Experimental data were fitted with hyperbolic function y = Pmax * x/(KI + x); (**c**) α-parameter related to the photosynthetic efficiency at limiting light intensity; (**d**) Oxygen consumption by dark respiration.

The ability of Cy5 to enhance diatom photosynthesis was also investigated by measuring the photosynthetic activity as light-dependent evolution of oxygen by cells, in an early exponential phase, grown in the presence and absence of Cy5 (Fig. 4a). The presence of Cy5 increases the maximum photosynthetic activity (Pmax) by ~ 49% versus the control sample (Fig. 4b). Similarly, the α-parameter, indicating the photosynthetic efficiency at limiting light intensity, was increased by ~ 48% (Fig. 4c). Interestingly, an increased photosynthetic O_2_ production was evident across all the irradiances range herein tested, with a ~ 50% increase in Cy5 treated cells (Fig. 4a inset). As reported in the supplementary Table 1, the chlorophyll content per cell was not significantly influenced by the presence of Cy5, so the increase in oxygen content was relevant on both chlorophyll and cell basis.

These results, together with the light-dependent effect of Cy5, supports the hypothesis that the dye generates photosynthetic enhancement. In fact, oxygen production is related to the water splitting photosynthetic mechanism that fuels electron transport chains; hence, the improved O_2_ production in the presence of Cy5 can be regarded as plausible proof of photosynthetic enhancement.

Moreover, oxygen consumption in the dark was similar in the presence and absence of Cy5, suggesting that there are no major effects of Cy5 on mitochondrial oxidative pathways (Fig. 4c). Increased photosynthetic activity was consistent with the increased growth rate.

### Location of the dye in the cells

Once the light-dependent effect of Cy5 on diatom growth had been assessed, confocal analysis was performed to investigate the location of the dye in Cy5 treated diatoms with respect to bare cell structures. Samples were obtained using diatoms incubated with Cy5 in the earlier stage (45 min) and in the growth plateau stage (8 days), to evaluate both the incorporation of Cy5 and cell morphology related to their viability.

**Figure 5:**
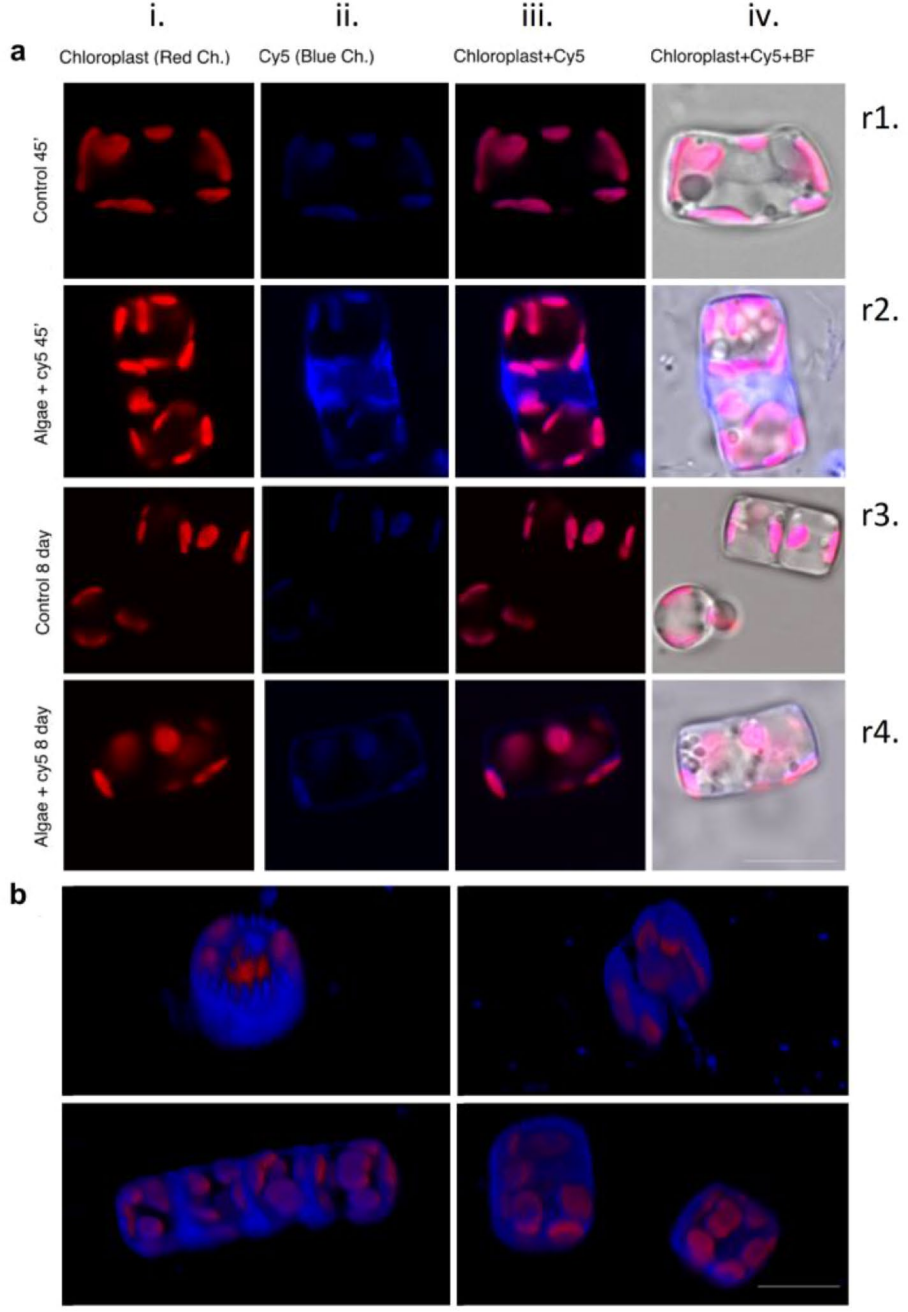
(**a**) Confocal microscopy image of diatom control (Ctrl), and diatoms grown with Cy5 1 μM after 45 min and 8 days’ incubation in normal lighting conditions (light: dark 16: 8 h). Red and blue colors were arbitrarily assigned for chloroplast and Cy5 emission, respectively. (Size bar 10 μm); (**b**) 3D reconstruction of diatoms incubated with Cy5 after 45 min (Size bar 10 μm).

Emission colors were arbitrarily assessed as red and blue for chloroplasts and Cy5, respectively, to distinguish their photoluminescence. Confocal microscopy images detected 45 min after incubation unequivocally showed the presence of Cy5 in diatom frustules (Fig. 5a: *ii.* Row 2) where chloroplasts appeared sticked (Fig. 5a: *i.* Row 2). Interference from the fluorescence of photosynthetic organelles in the Cy5 channel, also observed in the control sample (Fig. 5a: *ii*. Rows 1–4) did not allow the location of Cy5 in chloroplasts to be unambiguously assessed. The dye’s incorporation in cells was also confirmed observing Cy5 emission in the mitotic septum, as shown in Fig. 5a (*ii.* and *iii*. Row 2) and in the 3D reconstruction of Fig. 5b ^44^.

Confocal analysis was also performed after 8 days’ incubation with Cy5, *i.e.* in proximity of the growth plateau. A weak photoluminescence of Cy5 was observed in the cell walls (Fig. 5a: *ii.* and *iii*. Row 4), but diatoms’ morphology, as well as the location and intensity of chloroplasts’ luminescence (Fig. 5a: *ii*. and *iii*. Row 4) confirmed the viability of Cy5 treated microalgae versus the control (Fig. 5a: Rows 1 and 3). General morphological assessment using bright field microscopies showed that all diatoms, examined at 45 min and 8 days (Fig. 5a: *iv*. Rows 1–4) were able to produce similar box-like silica structures both in the presence and absence of Cy5.

Confocal microscopy images were also recorded for bare and Cy5 treated cells under darkness, in order to inhibit photosynthesis (Supplementary Fig. S2). The presence of Cy5 was still evident after 8 days but with a weaker signal and, after 8 days, diatoms and chloroplasts changed morphology due to the negative effects of prolonged darkness that inhibited photosynthesis. Anyway, the similar pattern of Cy5 distribution in cells either light treated or incubated in the dark demonstrates that the localization of Cy5 within the cells is not related to light availability.

### Time resolved fluorescence spectroscopy

**Figure 6:**
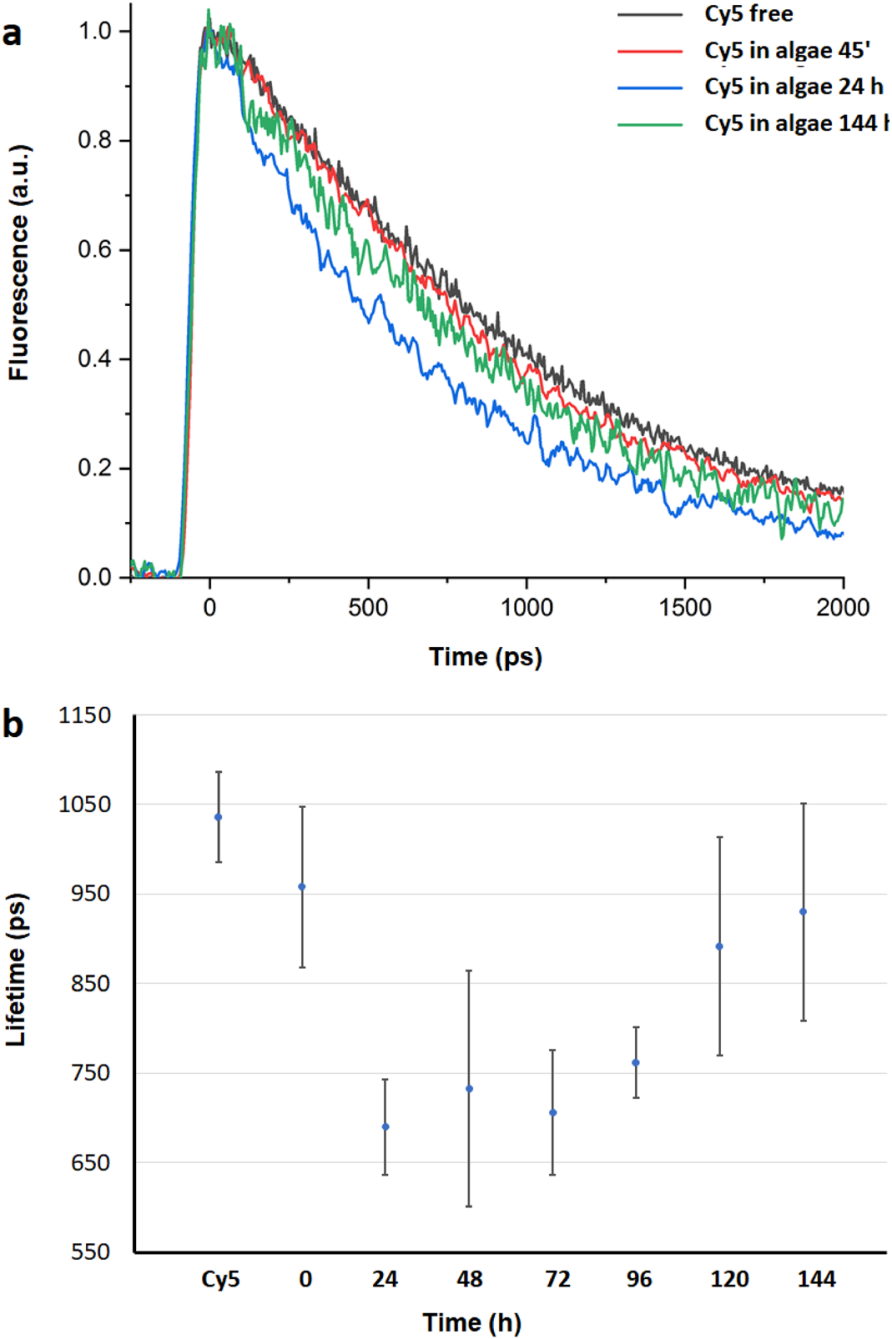
(**a**) Temporal evolution of Cy5 alone and inside diatoms at different incubation times. (**b**) Evolution of the fluorescence lifetime decay of Cy5 alone and incorporated into diatoms after different incubation times.

Interaction between Cy5 and the photosynthetic unit was investigated in vivo by time-resolved fluorescence spectroscopy measurements using a streak camera, as described in the Methods Section. Figure 6a and Supplementary S3 show the temporal decays obtained by integrating the fluorescence signal over the 625–665 nm spectral range, where the contribution of Cy5 is much higher compared to Chlorophyll, at different times after incubation of Cy5 with algae. In order to remove the free Cy5 (not incorporated into diatoms) we carried out three washing steps with centrifugation collection and addition of fresh sea water medium. Temporal decay of the molecular Cy5 dye in seawater was also recorded. Figure 6b shows the fluorescence lifetime obtained fitting, with a monoexponential decay function, the temporal profiles as a function of the incubation time of diatoms with Cy5. For all the samples we observed a monoexponential decay, indicating a single population of emitting molecules. This can be ascribed to the removal of free dye molecules, not incorporated, by the washing procedure.

Figure 6 b shows a decrease of Cy5 fluorescence lifetime after 24 h incubation and this keeps almost constant up to 96 h. Over longer time periods, a recovery of the fluorescence lifetime was detected but even at 144 h, the fluorescence decay was still faster compared to the measurements carried out after 45 min (time 0 in Fig. 6a). Lifetime decrease of Cy5 is consistent with the FRET process between Cy5 (donor) and Chl a (acceptor). Förster radius (R_*0*_), the distance at which energy transfer rate is 50%, for this donor acceptor pair is 5.1 nm^42^. The FRET yield of energy transfer E has been estimated by using the expression

$$  E = 1{-}\tau _{{DA}} /\tau _{D} ,  $$

where τ_DA_ and τ_D_ are the lifetimes of the donor (Cy5) in presence and absence of the acceptor (Chl), respectively. By comparing the Cy5 fluorescence lifetime in the presence and absence of diatoms (Fig. 6b), a FRET yield of energy transfer between donor and acceptor of ~ 30% and 10% was evaluated at 24 h and 144 h, respectively.

### Immunoblotting quantification of photosynthetic subunits

**Figure 7:**
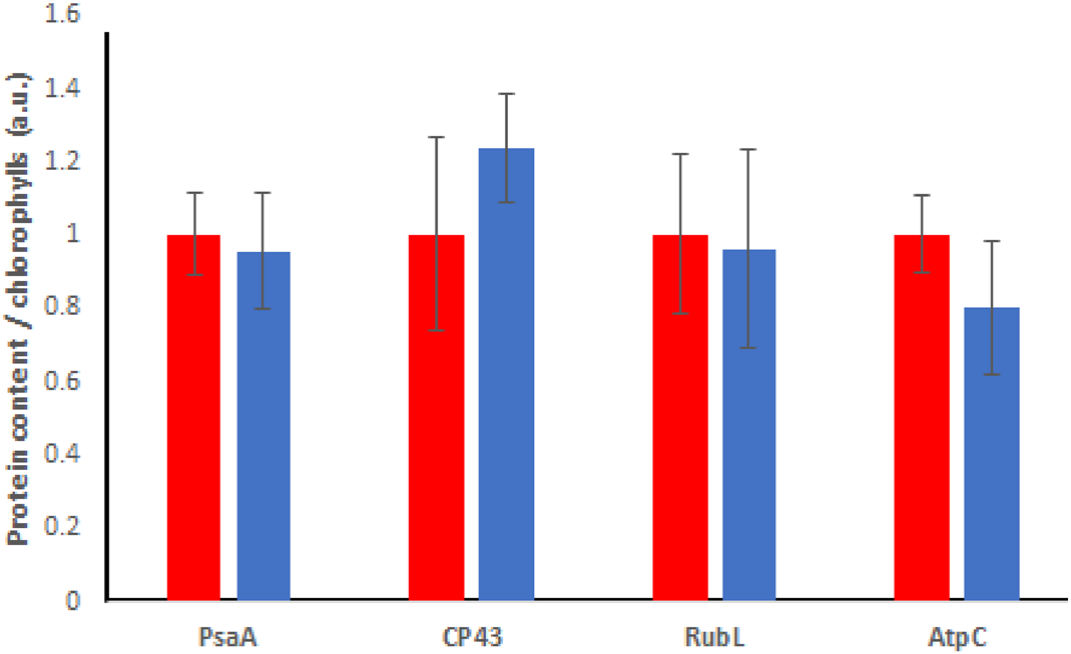
Immunoblotting quantification of photosynthetic subunits. The bands appearing upon western blot analysis were quantified by densitometry and normalized to the control case to determine the protein content in the Cy5 treated sample.

Immunoblotting analysis was performed with specific antibodies recognizing subunits of PSI (PsaA), PSII (CP43), chloroplast ATPase (ATPase C subunit) and RUBISCO (Fig. 7), in order to evaluate whether the increased photosynthetic efficiency observed in the presence of Cy5 could be related to an effect of Cy5 on the stoichiometry of the photosynthetic apparatus. Figure S4 in the Supplementary shows full-length blot. As reported, no major changes were observed for PSI, PSII, chloroplast ATPase or RUBISCO content on a chlorophyll basis in the presence or absence of Cy5. These results indicate that the increased light harvesting properties of the photosynthetic apparatus in the presence of Cy5 did not change the PSI/PSII or the ATPase or RUBISCO/chlorophylls ratios suggesting that the presence of Cy5 did not significantly affected the organization of the photosynthetic apparatus.

## Discussion

Effective ways of increasing the productivity and growth rate of diatoms have an important impact on their use as a source of biomaterials and biofuels^3^. The large-scale production of microalgae and related biocomponents can be facilitated by enhancing photosynthesis. The use of light-absorbing molecules to increase light collection in spectroscopic regions not covered by photosynthetic pigments of the microalgae represents a suitable and scalable approach which would rule out the need to use technologies based on genetic modification. A limited number of spectral shifting systems and dyes converting unabsorbed wavelength regions from white light sources into absorbable light have been reported in the literature ^33–35,45^. However, due to their toxicity, these dyes had to be maintained physically separated from the algal culture, acting, that is, just as spectral shifting solutions. This introduces complexity into the structure of bioreactors and reduces the industrial applicability of this approach.

Here we have described a cyanine molecule Cy5 (Fig. 1) functioning as an artificial antenna that can be easily incorporated in vivo into *Thalassiosira weissflogii* diatoms by simple addition to the culture medium, exploiting its amphiphilic properties for crossing cell membranes: Cy5 partially fills the diatom pigment absorbance gap in the orange spectral region, is non-toxic and leads to increased cells growth and biomass production (Figs. 1 and 3).

In normal white lighting conditions (70 µmol/m^2^ s), the addition of Cy5 leads to a ~ 23% increase in diatom biomass production (Fig. 3b) with a 40% increase in cell density (Fig. 3a) in a week. The enhanced cell density mediated by Cy5 was achieved using 1 μM dye concentration, which is significantly lower than that previously used for dyes spatially confined from cultures:^33^ 1 μM was selected as the suitable Cy5 concentration after the experiments reported in Supplementary S5.

The involvement of Cy5 in the photosynthetic pathway was demonstrated in a preliminary way by evaluating the consistent Cy5 light dependent effect on diatoms’ growth in different lighting conditions (Fig. 3). In fact, the growth of Cy5 treated diatoms was increased under a white light source that excites the Cy5 antenna (Fig. 3a). On the contrary, no effect was observed when diatom cells treated with Cy5 were grown in dark conditions (Fig. 3b) or on illumination with blue light (410–450 nm) which is not absorbed by Cy5 (Fig. 3d).

Under white lighting conditions, Cy5 was also found to promote lipid biosynthesis in diatoms: photosynthesis is strictly correlated with lipid metabolism in photosynthetic microorganisms and diatoms represent the major group of microalgae exploiting fixed inorganic carbon to produce lipidogenic masses, thus representing promising sources for biofuel production^46^. Lipidome has been already investigated in diatoms^43,47^, being composed of saturated and unsaturated C16 FFAs in high concentration. GC–MS spectrometry carried out after esterification reaction of free fatty acids extracted from lipidome of both pristine and Cy5 treated diatoms revealed a 12% increase of C16 FAMEs as an effect of the dye incorporation (Supplementary Fig. S1). The increased lipid productivity in Cy5 treated cells further reinforced the observed improved photosynthetic efficiency in the presence of Cy5, being lipids the macromolecule class with the highest content in chemical energy per gram.

Confocal microscopy images demonstrate the dye’s incorporation (Supplementary Fig. 5). The presence of the dye in cells, in close proximity to chloroplasts, is significant when attempting to bring about Cy5-Chl energy transfer.

Time-resolved fluorescence spectroscopy (Fig. 6 and Supplementary S3) reveals a decrease in Cy5 excited state lifetime over a period of days with a maximum after 24 h of incubation, suggesting that a FRET mechanism from Cy5 to Chl *a* pigments potentiating light harvesting is a plausible explanation for the increase observed in photosynthetic efficiency. After 24 h, the observed quenching of the incorporated Cy5 keeps almost constant up to 96 h and then it gradually decreases but it never reaches the start value, suggesting that energy transfer occurs over days. Slow Cy5 degradation over a period of days and consequent absorbance-emission decay can be held responsible for FRET rate fading over time, since reducing the donor concentration determines a consequent increase in donor–acceptor average distance.

To further investigate the mechanism of energy transfer from the dye to diatoms pigments, we also explored the effect of a 1 µM Cy5 solution acting as spectral shifter physically separated and interposed between the culture medium and the light source^33^. In this case, no effect on cells growth kinetics was observed (Supplementary Fig. S6), this supporting the hypothesis that incorporation allows the dye to act as a FRET donor in close proximity of diatoms plastid.

Oxygen production measurements provided further evidence of increased photosynthetic activity stimulated by Cy5 treatment (Fig. 4). The Pmax parameter (Fig. 4b), indicating the maximum oxygen produced by the Photosystem II of Cy5 stained diatoms, and the photosynthetic efficiency at limiting light conditions (α-parameter) were in both cases strongly increased in the presence of Cy5, with in general 50% increase of photosynthetic oxygen evolution in dye treated cells. Differently, no Cy5 effect was observed in dark respiratory conditions (Fig. 4d). Cy5 effect on O_2_ production occurs both at low and high light irradiance (Fig. 4a): as reported in the literature, this result may be due to a photosynthetic apparatus rearrangement to manage the increased excitation pressure at the level of the Photosystems due to the increased light harvesting due to Cy5 addition. Accordingly, several microalgae exhibited enhanced Pmax when grown at higher irradiances as a consequence of acclimation to these conditions. Similarly, strains selected for having a reduced chlorophyll content per cell, allowing for a better penetration of the light available in the photobioreactor, were in many cases characterized by an increased oxygen evolution not only in limiting light, but also in saturating light conditions^48–50^. In this case it is possible to consider Cy5 treated diatoms as cells experiencing an higher excitation pressure on Photosystems, likely inducing adaptation mechanisms in order to manage the increased photon flux ^51,52^. We evaluated the expression of proteins relating to photosynthesis after the incorporation of Cy5 for a week in normal lighting conditions. A similar organization of the photosynthetic apparatus was observed in presence or absence of Cy5 (Fig. 7 and Fig. S4). The possible adaptation mechanisms induced by Cy5 treatment do not include variations in the stoichiometry of photosystems or changes in the ATPase or RUBISCO content but they could be related to a more efficient production and consumption of ATP and NADPH. Oxygen evolution is related to the onset of a photosynthetic electron transport leading to NADPH formation coupled with proton transport from lumen to stroma to establish the electrochemical proton gradient used by the ATPase to produce ATP. Hence, improved light harvesting by Cy5 causes increased electron transport across the photosynthetic apparatus, as witnessed by increased light-dependent oxygen evolution compared to the control, leading to increased ATP and NADPH formation which can then be used by the carbon fixing reactions for the assimilation of CO_2_ into organic molecules. RUBISCO is a key enzyme for photosynthesis, whose activity is strongly controlled by the organism to manage metabolic activity. After 24 h incubation, the RUBISCO content was similar both in the presence and absence of Cy5, suggesting that the increased photosynthetic efficiency of Cy5 treated cells is due to increased production of ATP and NADPH during the photosynthetic light phase, which are then used by the Calvin-Benson cycle to fix CO_2_ in the biomass. It is important to note that the adaption of the chloroplast and cell metabolism to the increased excitation pressure on the photosynthetic apparatus could be complex: for instance, the increased lipid productivity observed in the Cy5 treated cells could be related to the need of increased regeneration of NADP^+^ to properly manage the increased photosynthetic electron transport caused by improved light harvesting. It is also worth noting that the use of a pigment being able to collect wavelengths poorly or not absorbed by chlorophylls, as in the case of Cy5, might contribute to mitigate the negative effect for biomass productivity caused by self-shading in highly pigmented cultures. Indeed, it is well known that self-shading is one of the main limitations for microalgae cultivation^49^, with the most of light wavelengths absorbable by chlorophylls being absorbed by the first layer of the cultures, leaving the inner layers almost in the dark or exposed to wavelength poorly absorbed by the photosynthetic apparatus. By using a dye as Cy5, or other dyes properly developed, it would be possible to collect these wavelengths not absorbed by chlorophylls, improving the overall light use efficiency of the cultures. To efficiently use Cy5 or other dyes for this purpose it will be important to set-up the dye concentration.

In conclusion, microalgae are highly attractive for industry as feedstock for food and pharmaceutics and as a source of lipids for biofuel production. However, the costs of achieving a high rate of growth, the difficulties in genetically modifying non model microalgal species for increasing growth, and the resistance of public opinion toward genetically modified organisms, limit their industrial use. In this work, we have demonstrated enhanced photosynthesis in diatom microalgae that increases biomass and growth without resorting to genetic modification. We stained living *Thalassiosira weissflogii* diatoms with a cyanine Cy5 dye, which partially fills the orange absorption gap of the photosynthetic pigments and increases light harvesting. Besides the enhanced growth and biomass, including lipid fraction, Cy5 increases diatom oxygen production. The most credited role for Cy5, demonstrated by spectroscopic measurements, is that it acts as an energy transfer donor for chlorophyll. Moreover, the energy transfer occurs on Chl *a*, which is a pigment present in most photosynthetic organisms. This approach may thus be in principle extended to other classes of photosynthetic organisms and can sustain diatoms and, in general, microalgae industrial valorization for biomass production to be further processed to obtain feed, food, bio-compounds or even potentially biofuels. The enhanced light response may boost algal growth by increasing the photoactive range in large incubators. In addition, the potential tuning of the spectral response makes it possible to envision the use of algae with any desired light spectrum, whether natural or artificial.

## Methods

### Algal culture conditions

The algal strain of *Thalassiosira weissflogii* diatoms (CCAP 1085/10, Scottish marine Institute, Scotland UK) was used. Diatoms were grown in F/2 Guillard-enriched seawater medium^53^ with 1:3000 of the stock sodium metasilicate, under sterile conditions in polystyrene 250 mL flasks. Before producing F/2 Guillard, seawater was sterilized in an autoclave and filtered twice (4–7 μm ø). Flasks were maintained in a photobioreactor under continuous fluorescent light (18 ± 2 °C, 65% ± 5% humidity, light: dark cycle 16:8 h, Pump Photon Flux: 70 µmol/m^2^ s).

### Pigments extraction and spectrophotometric analysis

In keeping with the literature^54^, 1 mL of washed algae was added in pigment extraction solution containing 8 mL of pure acetone, 1820 μL of distilled water, 180 μl of ammonium hydroxide 19%). The mixture was then centrifuged (5000 rpm for 15′) and 2 mL of pure hexane were added. The absorbance spectrum of pigments in hexane was evaluated by UV–visible spectrophotometer (Shimadzu UV-2401 PC).

### Diatom incubation with Cy5 and evaluation of cells growth and biomass

*Thalassiosira weissflogii* diatoms were added in polystyrene flasks containing freshly prepared F/2 Guillard enriched seawater medium (final diatom density of 5 × 10^4^ cell/mL; final flask volume 50 mL). For in vivo incorporation experiments, Cy5 dye was added once at the start point. A volume of 26 μL of Cy5 Stock commercial solution (0.0019 M Amersham Pharmacia Cyanine 5 NHS ester in DMSO, λ_exc_ = 640 nm, λ_em_ = 670 nm) were added to 50 mL of diatom cell culture, reaching 1 μM final Cy5 concentration. Diatom samples treated with the dye and used as control (Ctrl) bare cells were produced in quintuplicates. Algae were grown for 8 days in the photo-bioreactor, (18 °C ± 2, 65% ± 5% humidity, light: dark cycle 16:8 h), maintained at different light regimes. Growth in white light was achieved using 70 µmol/m^2^ s neon source conditions. The light path in the algal cultures was 3 cm. To evaluate cells growth using 0.5 and 2 μM concentrations of dye, experiments were carried on using the same experimental conditions.

Blue light (440–480 nm) was used at the same intensity and lighting cycle conditions used for white light. Diatom growth was evaluated daily by Thoma Chamber in contrast phase microscopy. Diatom density of each sample was calculated in triplicates and monitored for a week. According to literature, diatom growth rate was evaluated from 48 to 70 h of incubation, in the exponential growth phase. Biomass accumulation at the end of the growth curve was determined as dry weight per liter, as reported in the literature^22^.

### Lipid extraction and analysis

A set of three samples of pristine and Cy5 treated diatom cultures was used to perform analyses in triplicate. After incubation, cells were collected in centrifuge tubes (20′, 3500 rpm), and washed twice with a methanol: water solution (5 mL, 1:1 vol/vol). Pellets were dried overnight and 7 μL of standard solution of cis 2-hexadecenoic acid in ethanol (10 mg/mL) was added in each sample. Then, a Fatty Acid Methylation Kit (MAK224; Sigma Aldrich) was used to induce precipitation and separation of waxes and membrane lipids and to convert free fatty acids (FFA) into their methyl ester derivatives (FAME). To investigate the Cy5 effect on lipid biosynthesis in diatoms, we calculated the % area ratio (%R parameter)^55^ as it follows:

$$ \% R: \frac{Ame}{{As}}100 $$

where *Ame* is the area related to the diatoms C16 FAME (retention time: 15.9–16.1 min. range), As is the area under the internal standard peak (retention time: 15.45 min).

### Oxygen evolution curves

Oxygen evolution curves were performed as described in the literature^56,57^. Net oxygen production was calculated subtracting oxygen consumption in the dark after each measurement at different actinic lights. Experimental data were fitted with hyperbolic functions in order to retrieve the Pmax (maximum photosynthetic activity).

### Confocal microscopy

Diatom samples were washed in Milli-Q water. Diatoms were then pelleted and characterized by confocal laser scanning microscopy (LSM-510 confocal microscope, Zeiss). Microscopy configuration was: UV/Diode laser (λ_exc_ = 405 nm for chlorophyll, λ_exc_ = 640 nm for Cy5) and HC PL APO CS2 63x/1.40 Oil objective. Emission spectra were recorded in the visible spectral range (Δλ_em_ = 670–750 nm for chlorophyll, Δλ_exc_ = 650–670 nm for Cy5). Colors were arbitrarily assessed as blue for Cy5 and red for chloroplasts.

### Time resolved fluorescence spectroscopy

Time-resolved fluorescence measurements were carried out using a Ti: sapphire laser (Chameleon Ultra II, Coherent) with a repetition rate of 80 MHz and pulses with a temporal full width half maximum (FWHM) of ∼140 fs. The output was sent to an optical parametric oscillator (OPO) providing pulses in the NIR (1000–1400 nm). The signal was, then, doubled by beta barium borate crystal (BBO) to reach the final excitation light at 620 nm. A streak camera system (C5680, Hamamatsu), coupled to a spectrometer, was selected as the detection system giving spectro-temporal matrices with spectral and temporal resolutions of ∼1 nm and ∼20 ps, respectively. The fluorescence signal was separated from excitation light by a proper set of high pass filters.

### SDS-PAGE and immunoblotting

SDS-PAGE and immunoblotting were performed as described in the literature^54^ In details, Antibodies α-PsaA (AS06 172) α-CP43 (AS11 1787) α-ATPase C subunit (AS08 312) and α-RUBISCO large subunit (AS03 037) were obtained from Agrisera (https://www.agrisera.com/). Samples were loaded at different amount of chlorophylls (2; 1; 0.5, 0.25 µg) in order to evaluate the linearity of the signals detected. Western blots were digitalized by ChemiDoc MP imaging system (Bio-rad) and quantified by using Image Lab software from Bio-rad (https://www.bio-rad.com/it-it/product/image-lab-software?ID=KRE6P5E8Z).

### Statistics

A one-way ANOVA test was performed to evaluate the significance in difference between samples. Data were considered statistically significant for *P* < 0.05.

## Supplementary Information

Supplementary information.

## References

[1] Priyadarshani I, Rath B (2012). Commercial and industrial applications of micro algae–a review. J. Algal. Biomass Utln..

[2] Sayre R (2010). Microalgae: the potential for carbon capture. Bioscience.

[3] Lavaud J, Rousseau B, Etienne AL (2004). General features of photoprotection by energy dissipation in planktonic diatoms (Bacillariophyceae*)*. J. Phycol..

[4] Yang W, Lopez PJ, Rosengarten G (2011). Diatoms: self assembled silica nanostructures, and templates for bio/chemical sensors and biomimetic membranes. Analyst.

[5] Field CB, Behrenfeld MJ, Randerson JT, Falkowski P (1998). Primary production of the biosphere: integrating terrestrial and oceanic components. Science.

[6] Rabosky DL, Sorhannus U (2009). Diversity dynamics of marine planktonic diatoms across the Cenozoic. Nature.

[7] Falkowski PG, Katz ME, Knoll AH, Quigg A, Raven JA, Schofield O, Taylor FJR (2004). The evolution of modern eukaryotic phytoplankton. Science.

[8] Hildebrand M, Davis AK, Smith SR, Traller JC, Abbriano R (2012). The place of diatoms in the biofuels industry. Biofuels.

[9] Cicco SR, Vona D, Leone G, De Giglio E, Bonifacio MA, Cometa S, Fiore S, Palumbo F, Ragni R, Farinola GM (2019). In vivo functionalization of diatom biosilica with sodium alendronate as osteoactive material. Mater. Sci. Eng. C.

[10] Ragni R, Cicco SR, Vona D, Farinola GM (2018). Multiple routes to smart nanostructured materials from diatom microalgae: a chemical perspective. Adv. Mater..

[11] Cicco SR, Vona D, De Giglio E, Cometa S, Mattioli-Belmonte M, Palumbo F, Ragni R, Farinola GM (2015). Chemically modified diatoms biosilica for bone cell growth with combined drug-delivery and antioxidant properties. ChemPlusChem.

[12] Leone G, Vona D, Presti ML, Urbano L, Cicco S, Gristina R, Palumbo F, Ragni R, Farinola GM (2017). Ca 2+-in vivo doped biosilica from living Thalassiosira weissflogii diatoms: investigation on Saos-2 biocompatibility. MRS Adv..

[13] Ragni R, Scotognella F, Vona D, Moretti L, Altamura E, Ceccone G, Mehn D, Cicco SR, Palumbo F, Lanzani G, Farinola GM (2018). Hybrid photonic nanostructures by in vivo incorporation of an organic fluorophore into diatom algae. Adv. Funct. Mater..

[14] Ragni R, Cicco S, Vona D, Leone G, Farinola GM (2017). Biosilica from diatoms microalgae: smart materials from bio-medicine to photonics. J. Mater. Res..

[15] Ragni, R. Cicco, S. R., Vona, D. & Farinola, G. M. Nanostructured Silica from Diatoms Microalgae: Smart Materials for Photonics and Electronics. In *Green Materials for Electronics* (ed. Irimia-Valdu, M., Glowacky, E. D., Sariciftci, N. S. & Bauer, S.) 287–315 (Wiley-VCH, 2017).

[16] Trentacoste EM, Shrestha RP, Smith SR, Glé C, Hartmann AC, Hildebrand M, Gerwick WH (2013). Metabolic engineering of lipid catabolism increases microalgal lipid accumulation without compromising growth. PNAS.

[17] Kirschbaum MU (2011). Does enhanced photosynthesis enhance growth? Lessons learned from CO_2_ enrichment studies. Plant Physiol..

[18] Terry KL (1986). Photosynthesis in modulated light: quantitative dependence of photosynthetic enhancement on flashing rate. Biotech. Bioeng..

[19] Mann JE, Myers J (1968). Photosynthetic enhancement in the diatom Phaeodactylum tricornutum. Plant Physiol..

[20] Schofield O, Bidigare RR, Prézelin BB (1990). Spectral photosynthesis, quantum yield and blue-green light enhancement of productivity rates in the diatom Chaetoceros gracile and the prymnesiophyte Emiliania huxleyi. Mar. Ecol. Prog. Ser..

[21] Stephenson PG, Moore CM, Terry MJ, Zubkov MV, Bibby TS (2011). Improving photosynthesis for algal biofuels: toward a green revolution. Trends Biotech..

[22] Berteotti S, Ballottari M, Bassi R (2016). Increased biomass productivity in green algae by tuning non-photochemical quenching. Sci. Rep..

[23] Mooij T, Janssen M, Cerezo-Chinarro O, Mussgnug J, Kruse O, Ballottari M, Bassi R, Bujaldon S, Wollman F, Wijffels R (2015). Antenna size reduction as a strategy to increase biomass productivity: a great potential not yet realized. J. Appl. Phycol..

[24] Melis A (2009). Solar energy conversion efficiencies in photosynthesis: minimizing the chlorophyll antennae to maximize efficiency. Plant Sci..

[25] Fu W, Chaiboonchoe A, Khraiwesh B, Sultana M, Jaiswal A, Jijakli K, Nelson DR, Al-Hrout A, Baig B, Amin A, Salehi-Ashtiani K (2017). Intracellular spectral recompositioning of light enhances algal photosynthetic efficiency. Sci. Adv..

[26] Banerjee A, Banerjee C, Negi S, Chang JS, Shukla P (2018). Improvements in algal lipid production: a systems biology and gene editing approach. Crit. Rev. Biotechnol..

[27] Jeon S, Lim JM, Lee HG, Shin SE, Kang NK, Park YI, Hee-Mock Oh, Won-Joong J, Byeong-ryool J, Chang YK (2017). Current status and perspectives of genome editing technology for microalgae. Biotechnol. Biofuels.

[28] Ng IS, Tan SI, Kao PH, Chang YK, Chang JS (2017). Recent developments on genetic engineering of microalgae for biofuels and bio-based chemicals. Biotech. J..

[29] Milano F, Tangorra RR, Hassan Omar O, Ragni R, Operamolla A, Agostiano A, Farinola GM, Trotta M (2012). Enhancing the light harvesting capability of a photosynthetic reaction center by a tailored molecular fluorophore. Ang. Chemie Int. Ed..

[30] Hassan OO, La Gatta S, Tangorra RR, Milano F, Ragni R, Operamolla A, Argazzi R, Chiorboli C, Agostiano A, Trotta M, Farinola GM (2016). Synthetic antenna functioning as light harvester in the whole visible region for enhanced hybrid photosynthetic reaction centers. Bioconj. Chem..

[31] Sissa C, Painelli A, Terenziani F, Trotta M, Ragni R (2020). About the origin of the large Stokes shift in aminoalkyl substituted heptamethine cyanine dyes. Phys. Chem. Chemical Phys..

[32] La Gatta S, Milano F, Farinola GM, Agostiano A, Di Donato M, Lapini A, Foggi P, Trotta M, Ragni R (2019). A highly efficient heptamethine cyanine antenna for photosynthetic Reaction Center: from chemical design to ultrafast energy transfer investigation of the hybrid system. BBA Bioenerg..

[33] Prokop A, Quinn MF, Fekri M, Murad M, Ahmed SA (1984). Spectral shifting by dyes to enhance algae growth. Biotech. Bioenerg..

[34] Burak H, Dunbar A, Gilmour DJ (2019). Enhancement of Dunaliella salina growth by using wavelength shifting dyes. J. Appl. Phycol..

[35] Sung MG, Han JI, Lee B, Chang YK (2018). Wavelength shift strategy to enhance lipid productivity of Nannochloropsis gaditana. Biotechnol. Biofuels.

[36] Li CW, Chu S, Lee M (1989). Characterizing the silica deposition vesicle of diatoms. Protoplasma.

[37] Kucki M, Fuhrmann-Lieker T (2011). Staining diatoms with rhodamine dyes: control of emission colour in photonic biocomposites. J. R. Soc. Int..

[38] Weber F (1937). Assimilationsfaehigkeit und Doppelbrechung der Chloroplasten. Protoplasma.

[39] Strugger S (1938). Die Vitalfärbung des Protoplasmas mit Rhodamin B und 6 G. Protoplasma.

[40] Gundlach K, Werwie M, Wiegand S, Paulsen H (2009). Filling the “green gap” of the major light-harvesting chlorophyll a/b complex by covalent attachment of Rhodamine Red. BBA Bioenerg..

[41] Mantoura RFC, Llewellyn CA (1983). The rapid determination of algal chlorophyll and carotenoid pigments and their breakdown products in natural waters by reverse-phase high-performance liquid chromatography. Anal. Chimica Acta.

[42] Yacobi YZ (2012). From Tswett to identified flying objects: A concise history of chlorophyll a use for quantification of phytoplankton. Israel J. Plant Sci..

[43] Suroy M, Moriceau B, Boutorh J, Goutx M (2014). Fatty acids associated with the frustules of diatoms and their fate during degradation-A case study in *Thalassiosira weissflogii*. Oceanogr. Res. Pap..

[44] Desclés J, Vartanian M, El Harrak A, Quinet M, Bremond N, Sapriel G, Bibette G, Lopez PJ (2008). New tools for labeling silica in living diatoms. New Phytol..

[45] Wondraczek L, Batentschuk M, Schmidt MA, Borchardt R, Scheiner S, Seemann B, Schweizer P, Brabec CJ (2013). Solar spectral conversion for improving the photosynthetic activity in algae reactors. Nat. Commun..

[46] Hildebrand M, Davis AK, Smith SR, Traller JC, Abbriano R (2012). The place of diatoms in the biofuels industry. Biofuels.

[47] Pratoomyot J, Srivilas P, Noiraksar T (2005). Fatty acids composition of 10 microalgal species. Songklanakarin J. Sci. Technol.

[48] Perin J, Bellan A, Segalla A, Meneghesso A, Alboresi A, Tomas M (2015). Generation of random mutants to improve light-use efficiency of Nannochloropsis gaditana cultures for biofuel production. Biotechnol. Biofuels.

[49] Kirst H, Gines García-Cerdán J, Zurbriggen AS, Melis A (2012). Assembly of the light-harvesting chlorophyll antenna in the green alga Chlamydomonas reinhardtii requires expression of the TLA2-CpFTSY gene. Plant Physiol..

[50] Cazzaniga S, DallOsto L, Szaub J, Scibilia L, Ballottari M, Purton S, Bassi R (2014). Domestication of the green alga Chlorella sorokiniana: reduction of antenna size improves light-use efficiency in a photobioreactor. Biotechnol. Biofuels.

[51] Bonente G, Pippa S, Castellano S, Bassi R, Ballottari M (2012). Acclimation of Chlamydomonas reinhardtii to different growth irradiances. J. Bio. Chem..

[52] Costa BS, Jungandreas A, Jakob T, Weisheit W, Mittag M, Wilhelm C (2013). Blue light is essential for high light acclimation and photoprotection in the diatom Phaeodactylum tricornutum. J. Exp. Bot..

[53] Vona D, Cicco SR, Ragni R, Leone G, Presti ML, Farinola GM (2018). Biosilica/polydopamine/silver nanoparticles composites: new hybrid multifunctional heterostructures obtained by chemical modification of *Thalassiosira weissflogii* silica shells. MRS Commun..

[54] Calvano CD, Ventura G, Cataldi TR, Palmisano F (2015). Improvement of chlorophyll identification in foodstuffs by MALDI ToF/ToF mass spectrometry using 1, 5-diaminonaphthalene electron transfer secondary reaction matrix. Anal. Bioanal. Chem..

[55] Casiello M, Catucci L, Fracassi F, Fusco C, Laurenza AG, Di Bitonto L, Pastore C, D’Accolti L, Nacci A (2019). ZnO/ionic liquid catalyzed biodiesel production from renewable and waste lipids as feedstocks. Catalyst.

[56] Perozeni F, Stella G, Ballottari M (2018). LHCSR expression under HSP70/RBCS2 promoter as a strategy to increase productivity in microalgae. Int. J. Mol. Sci..

[57] Bonente G, Ballottari M, Morosinotto T-B, Ahn TK, Fleming GR, Niyogi KK, Bassi R (2011). Analysis of LhcSR3, a protein essential for feedback de-excitation in the green alga *Chlamydomonas* reinhardtii. Plos Biol..

