# Supplementary material for: Incorporating a molecular antenna in diatom microalgae cells enhances photosynthesis
Source: Sci Rep. 2021 Mar 4;11:5209. doi: 10.1038/s41598-021-84690-z (PMC7933240; doi:10.1038/s41598-021-84690-z)
Supplement: Supplementary file 1 — Supplementary information. [file 41598_2021_84690_MOESM1_ESM.docx]

**SUPPLEMENTARY MATERIAL**

Incorporating a molecular antenna in diatom microalgae cells enhances photosynthesis

Gabriella Leone,^1,2^ Gabriel De la Cruz Valbuena,^3^ Stefania Roberta Cicco,^4^ Danilo Vona,^1^ Emiliano Altamura,^1^ Roberta Ragni,^1^ Egle Molotokaite,^2^ Michela Cecchin,^5^ Stefano Cazzaniga,^5^ Matteo Ballottari,^5^ Cosimo D’Andrea,^2,3^ Guglielmo Lanzani^2,3^* and Gianluca Maria Farinola^1^*

^1^ Dipartimento di Chimica, Università degli Studi di Bari “Aldo Moro”, via Orabona 4, 70126 Bari (Italy).

^2^ Istituto Italiano di Tecnologia, Center for Nano Science and Technology, via Pascoli 70/3, 20133 Milano (Italy).

^3^ Dipartimento di Fisica, Politecnico di Milano, piazza Leonardo da Vinci, 32, 20133 Milano (Italy).

^4^ CNR-ICCOM, Dipartimento di Chimica, Università degli Studi di Bari “Aldo Moro”, via Orabona 4, 70126 Bari (Italy).

^5^ Dipartimento di Biotecnologie, Università degli studi di Verona, [Ca' Vignal 1, 37134 Verona](http://www.dbt.univr.it/?ent=luogo&id=133) (Italy)

* Corresponding should be addressed to [gianlucamaria.farinola@uniba.it](mailto:gianlucamaria.farinola@uniba.it); guglielmo.lanzani@polimi.it

**S1. Extraction and detection of lipids from both pristine and Cy5 treated diatoms and chlorophyll content determination**

As reported in the Method Section, cis 2-hexadecenoic acid (10 mg/mL stock solution in ethanol was added, as the internal standard, to triplicate samples of both pristine and Cy5 treated diatoms. Values of % R, for pristine and Cy5 treated cells, are reported in Fig. S1a. Fig. S1b shows (*i*) the GC-MS chromatogram from a fatty acids-negative control (flat brown line), (*ii*) the C16 FAME peak recorded for the internal reference (black line, 15.45 min.), and (*iii*) for a sample extracted from pristine cells (pink areas) in absence of the internal reference: pristine diatoms exhibit both unsaturated and saturated C16 FAME GC peaks at retention times 15.3 min and 15.9 min, respectively. Chromatograms of samples from (*iv*) pristine (red area) and (*v*) Cy5 treated diatoms (blue area) after addition of the internal standard (black area), are also reported in Fig. S1b. % R values were calculated from areas of saturated autogenic C16 FAME (blue area for Cy5 treated diatoms and red area for pristine diatoms) versus the internal reference peak (black area). The lipid evaluation revealed a 12% increase in the production of autogenic C16 FFAs due to an effect of Cy5 incorporation. Hence, an activation of microalgae metabolism occurs by Cy5 treatment. Anova elaboration correlated data with a p value < 0.1.


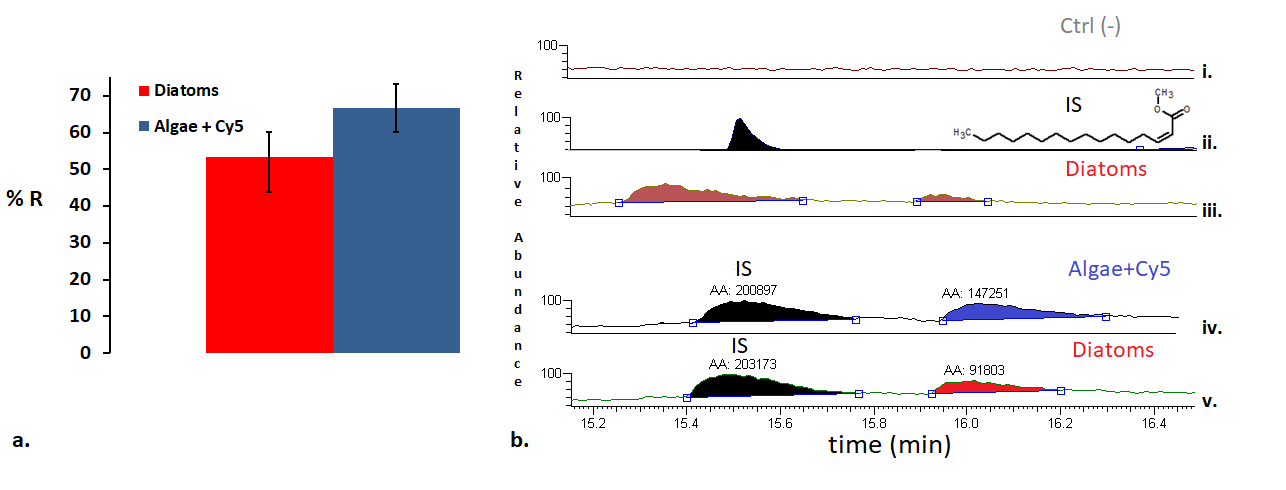


**Figure S1.** (a) Area ratio (% R) parameter and (b) C16 FAME peaks in GC-MS chromatograms of extracts from pristine diatoms and Cy5 diatoms. (*i*) a fatty acids-negative control (flat brown line); (*ii*) C16 FAME peak recorded for the internal reference (black line, 15.45 min.);(*iii*) sample from pristine diatoms (in the absence of internal reference) exhibiting both unsaturated (15.3 min.) and saturated (15.9 min.) C16 FAME GC peaks (pink areas); (*iv*) sample from Cy5 treated diatoms (blue area) after addition of the internal standard (black area); (*v*) sample from pristine diatoms (red area) after addition of the internal standard.

For the chlorophyll content determination, the methodology has been already reported in the main text (Methods section; Pigments extraction and spectrophotometric analysis). Table 1 reports the content of the pigment (pg) per cell for both untreated diatoms and treated diatoms with Cy5.

|  | chl pg/cell |
| --- | --- |
| Diatoms | 4.85 ± 0.62 |
| Algae+Cy5 | 3.88 ± 0.89 |

**Table 1**. Chlorophyll content per cell of *T. weissflogii* treated (ALGAE+CY5) and untreated (ALGAE) with Cy5 at the end of the growth curved reported in Figure 3.

**S2. Confocal microscopy of diatoms incubated with Cy5 under dark**

Confocal images of diatoms grown under dark, without performing photosynthesis are shown in Fig. S2. Samples were produced as described in the main text. Under normal lighting conditions, the presence of Cy5 was still evident after 8 days but with a weaker signal due to the degradation of the dye. After 8 days, diatoms and chloroplasts changed morphology due to the negative effects of prolonged darkness on *Thalassiosira weissflogii* cultures.


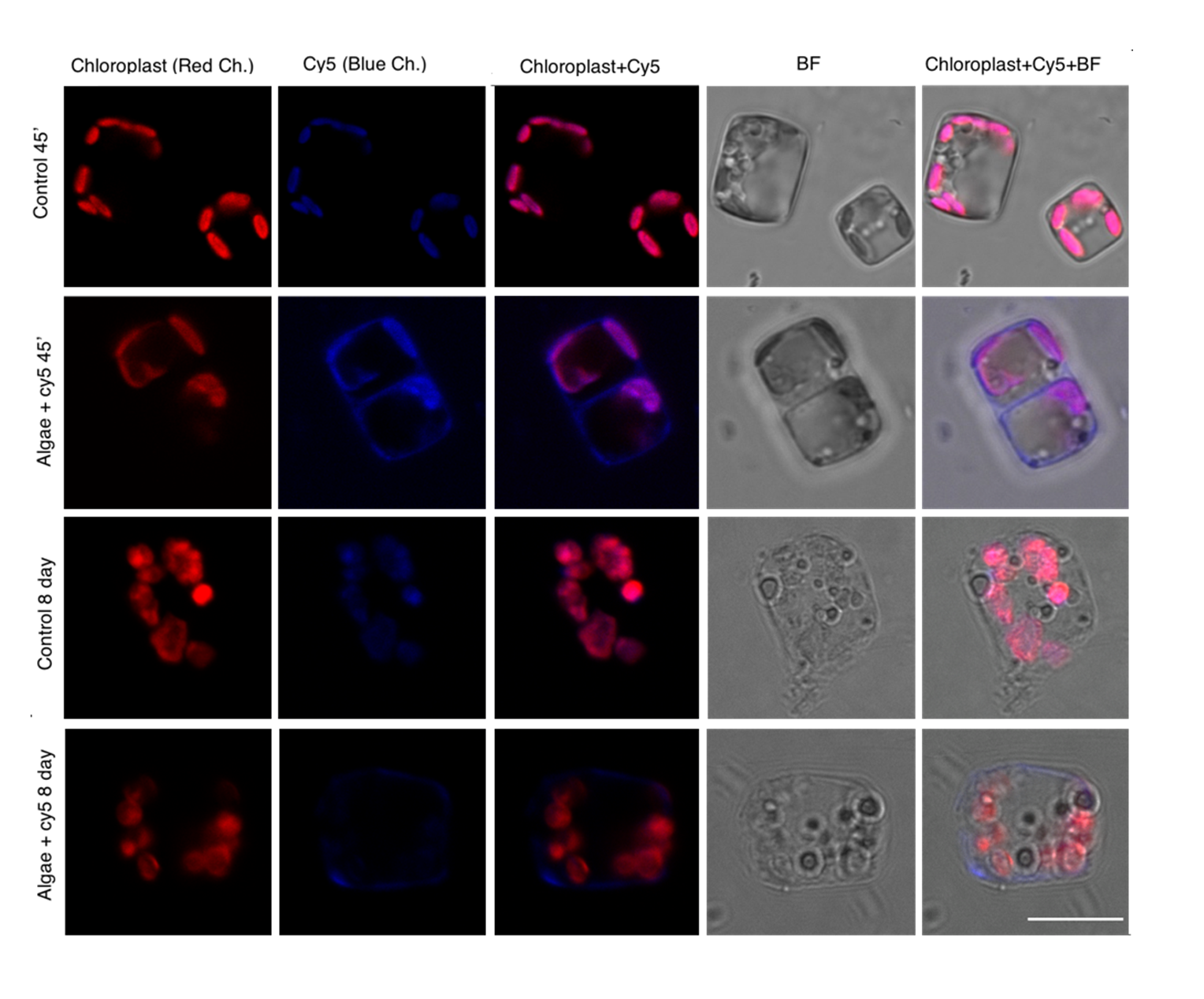


**Figure S2.** Confocal microscopy of diatom control (Ctrl), and diatoms grown with Cy5 1 µM after 45 minutes and 8 days of incubation in darkness, avoiding photosynthesis. *(*Size bar 10 µm).

**S3. Time-resolved fluorescence spectroscopy of Cy5 treated diatoms**

Time-resolved fluorescence spectroscopy measurements (Fig. S3) were performed every day over a week on living pigmented diatoms grown with Cy5 and the temporal decay was compared to the one of Cy5 free in solution. The maximum quenching occurs after 24 hours of incubation and it is preserved until 96 hours. For longer times an increase of the fluorescence lifetime was observed. These evidences support the hypothesis of energy transfer among Cy5 and chlorophyll *a*.


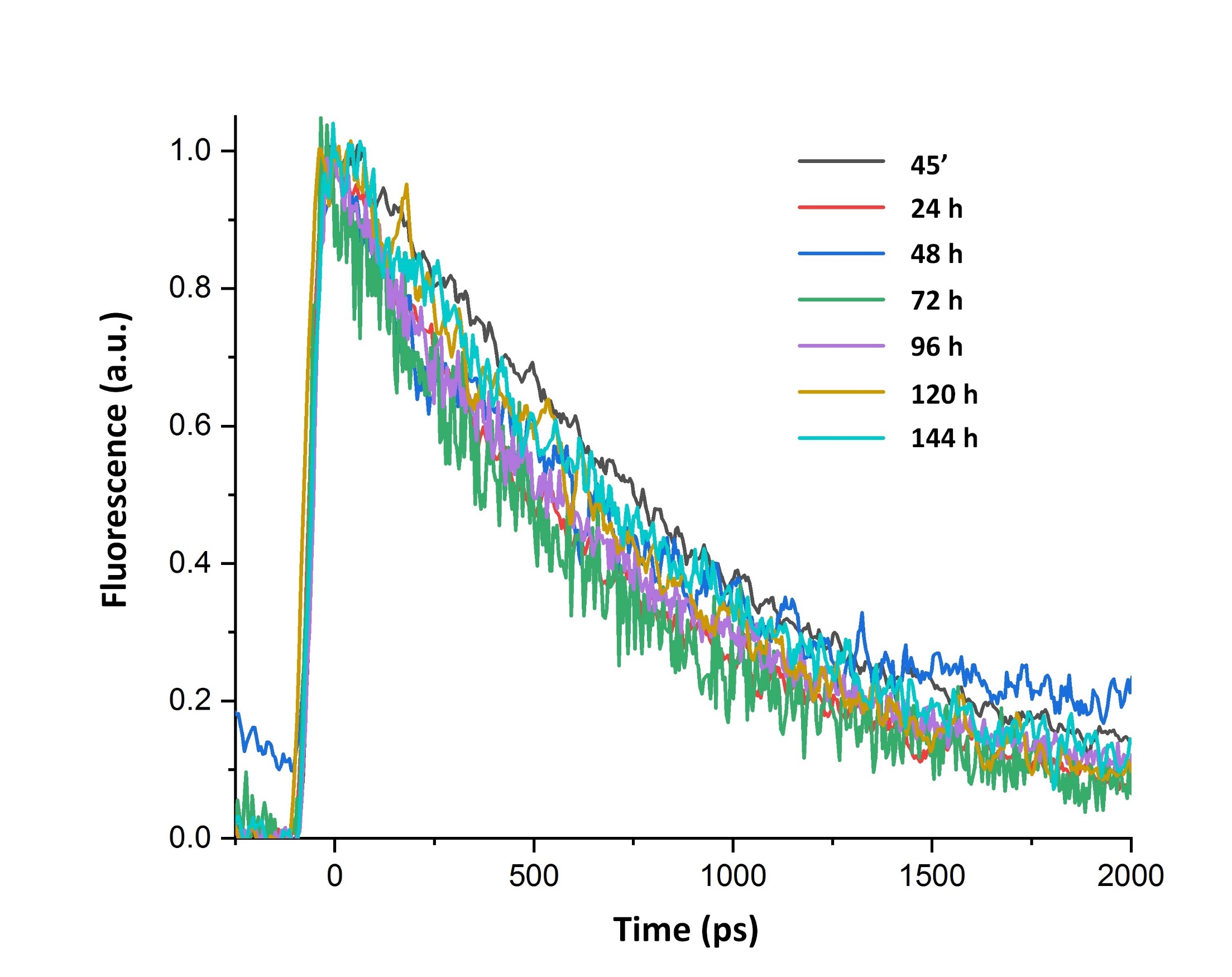


**Figure S3.**  Temporal evolution of photoluminescence of Cy5 alone and inside living diatoms at different incubation times.

**S4. Protein expression**

Photosynthetic protein expression of bare and Cy5 treated diatoms (Fig. S4) was monitored by immunoblotting analysis. Antibodies α-PsaA (AS06 172) α-CP43 (AS11 1787) α-ATPase C subunit (AS08 312) and α-RUBISCO large subunit (AS03 037) were obtained from Agrisera (https://www.agrisera.com/). Samples were loaded at different amount of chlorophylls (2; 1; 0.5, 0.25 µg) in order to evaluate the linearity of the signals detected. Western blots were digitalized by ChemiDoc MP imaging system (Bio-rad) and quantified by using Image Lab software from Bio-rad (https://www.bio-rad.com/it-it/product/image-lab-software?ID=KRE6P5E8Z). As mentioned in the main text, no difference of PSA, CP43, ATPase C and RUBISCO amounts was observed between the control and the Cy5 treated sample.


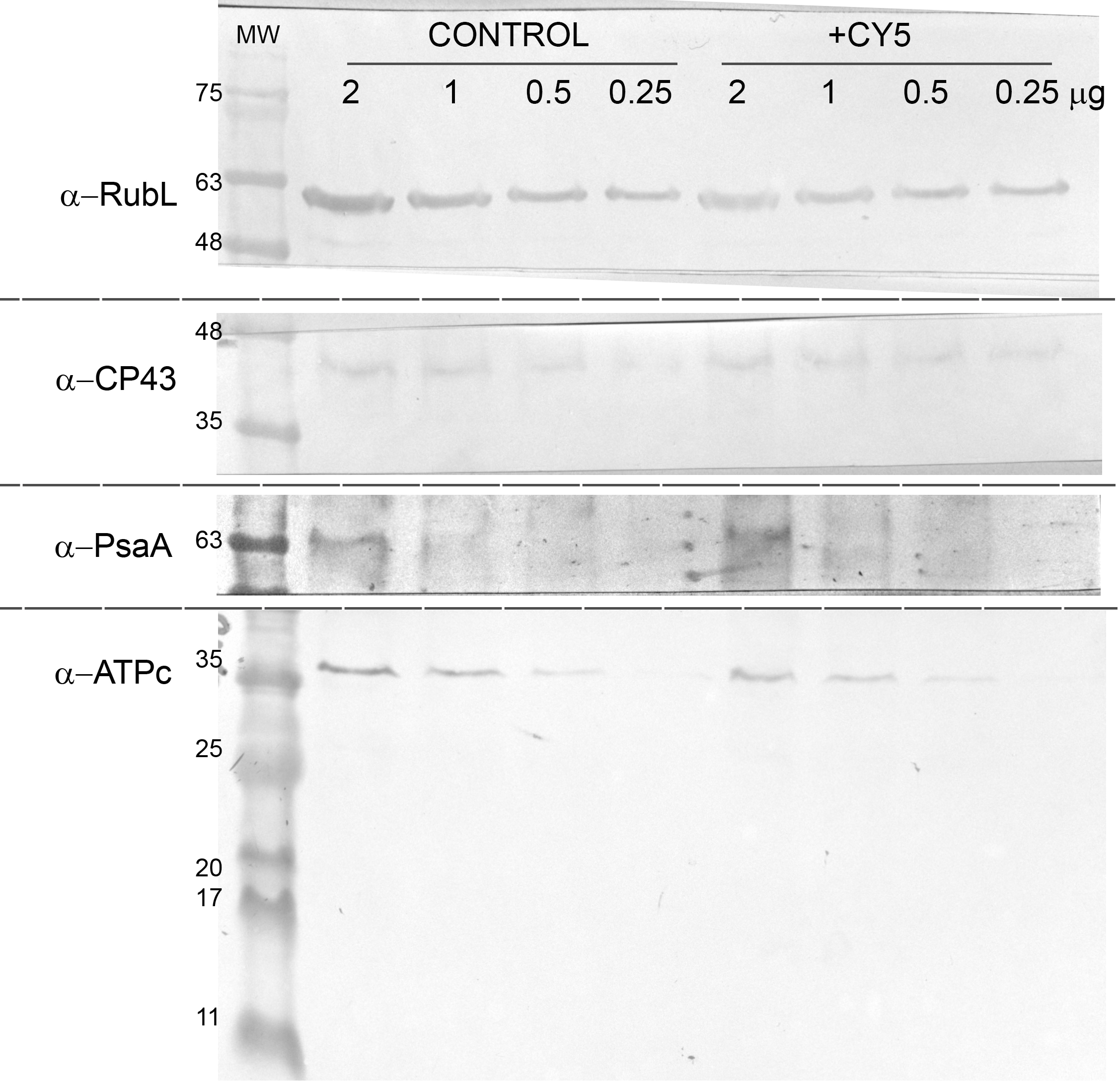


**Figure S4.** Immunoblotting quantification of photosynthetic subunits. Specific antibodies recognizing the large subunits of RUBISCO (RubL), CP43, PsaA and the C subunit of chloroplastic ATPase (ATPC) were used to perform immunoblotting reactions. Samples were loaded at different amount of chlorophylls (2; 1; 0.5, 0.25 µg) as reported on the top of the descriptive Figure. The different nitrocellulose filters used for the different immunoblotting reactions are reported separated by black lines. Pre-stained molecular weight (MW) marker was used as reported in the Figure.

**S5. Effects of Cy5 concentration on cells density**

The effects on cell density of different concentrations of Cy5 were investigated using 0.5, 1 and 2 μM solutions of the dye. Cells were grown in normal lighting conditions (light:dark cycle 16:8 h, Pump Photon Flux: 70 µmol/m^2^s). Figure S5 shows percentages of increase of cells densities after 7 days of Cy5 incubation versus control pristine diatoms. The growth kinetics increased only using 1 μM Cy5 solution, whereas 0.5 and 2 μM solutions of the dye did not alter the cells growth with respect to the control. 0.5 μM concentration is likely not sufficiently high to enhance light harvesting, while the dark blue colored 2 μM solution of the dye is thought to reduce light transmittance in the cell medium, having optical shielding effect on light absorption by cells.


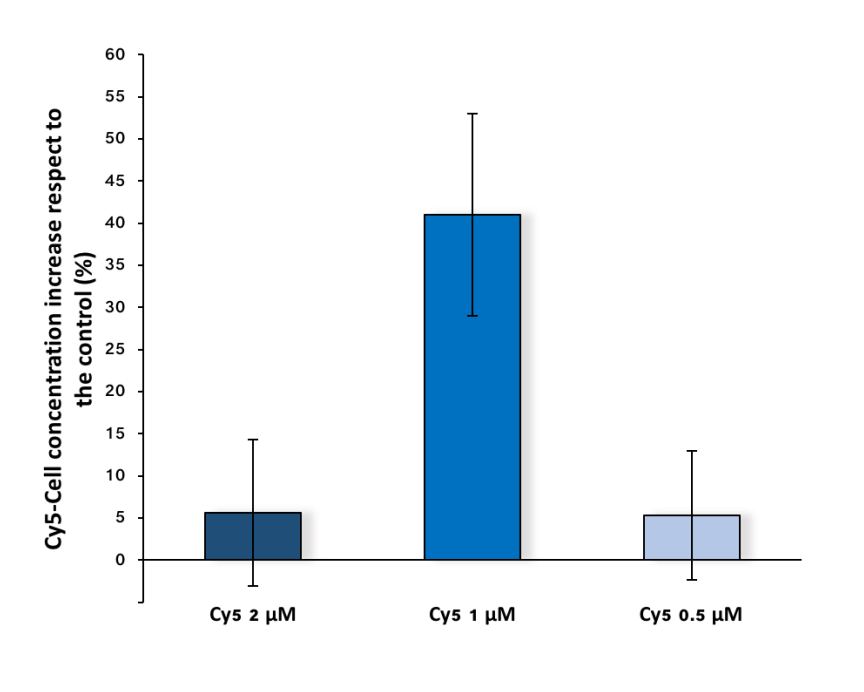


**Figure S5.** Cells density after 7 days for diatoms treated with (0.5, 1 and 2 μM) Cy5 solutions versus bare control microalgae**.**

**S6 Effect on diatom growth of Cy5 when maintained separated from the cell**

**
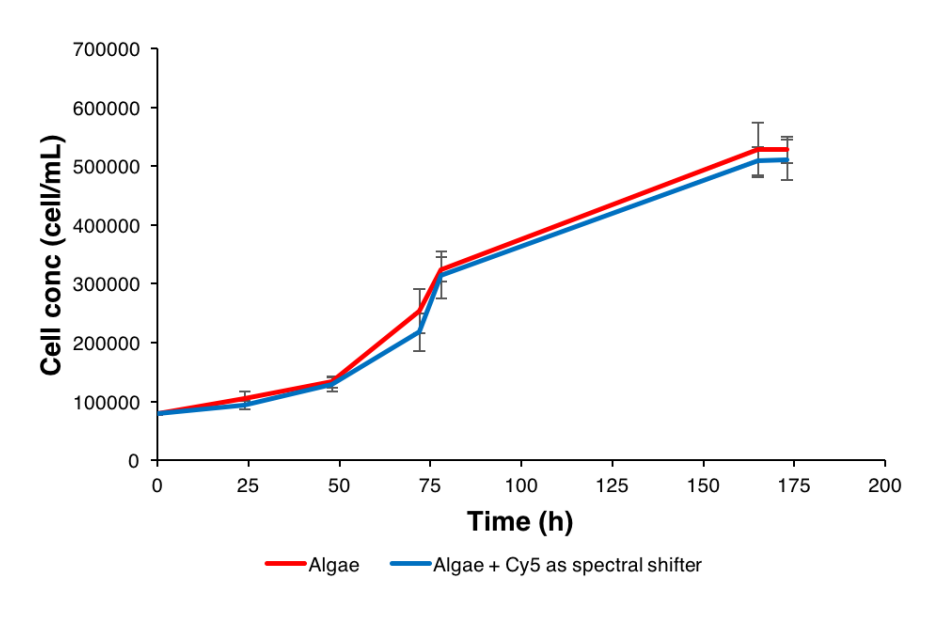
**

**Figure S6.** Kinetics of cells growth using 1 μM Cy5 as spectral shifter physically separated from diatoms.
